# Validation of diagnostic codes and epidemiologic trends of Huntington disease: a population-based study in Navarre, Spain

**DOI:** 10.1186/s13023-021-01699-3

**Published:** 2021-02-10

**Authors:** Esther Vicente, Ainara Ruiz de Sabando, Fermín García, Itziar Gastón, Eva Ardanaz, María A. Ramos-Arroyo

**Affiliations:** 1 Community Health Observatory Section, Instituto de Salud Pública y Laboral de Navarra, IdiSNA (Navarre Institute for Health Research), Pamplona, Spain; 2 Department of Health Sciences, Universidad Pública de Navarra, IdiSNA, Pamplona, Spain; 3 Department of Medical Genetics, Complejo Hospitalario de Navarra, IdiSNA, Pamplona, Spain; 4 Fundación Miguel Servet-Navarrabiomed, IdiSNA, Pamplona, Spain; 5 Department of Neurology, Complejo Hospitalario de Navarra, IdiSNA, Pamplona, Spain; 6 CIBER of Epidemiology and Public Health (CIBERESP), Madrid, Spain

**Keywords:** Huntington disease, Incidence, Mortality, Prevalence, Trends, Diagnostic codes, Positive predictive value, Sensitivity, Rare Diseases Registry

## Abstract

**Background:**

There is great heterogeneity on geographic and temporary Huntington disease (HD) epidemiological estimates. Most research studies of rare diseases, including HD, use health information systems (HIS) as data sources. This study investigates the validity and accuracy of national and international diagnostic codes for HD in multiple HIS and analyses the epidemiologic trends of HD in the Autonomous Community of Navarre (Spain).

**Methods:**

HD cases were ascertained by the Rare Diseases Registry and the reference Medical Genetics Centre of Navarre. Positive predictive values (PPV) and sensitivity with 95% confidence intervals (95% CI) were estimated. Overall and 9-year periods (1991–2017) HD prevalence, incidence and mortality rates were calculated, and trends were assessed by Joinpoint regression.

**Results:**

Overall PPV and sensitivity of combined HIS were 71.8% (95% CI: 59.7, 81.6) and 82.2% (95% CI: 70.1, 90.4), respectively. Primary care data was a more valuable resource for HD ascertainment than hospital discharge records, with 66% versus 50% sensitivity, respectively. It also had the highest number of “unique to source” cases. Thirty-five per cent of HD patients were identified by a single database and only 4% by all explored sources. Point prevalence was 4.94 (95% CI: 3.23, 6.65) per 100,000 in December 2017, and showed an annual 6.1% increase from 1991 to 1999. Incidence and mortality trends remained stable since 1995–96, with mean annual rates per 100,000 of 0.36 (95% CI: 0.27, 0.47) and 0.23 (95% CI: 0.16, 0.32), respectively. Late-onset HD patients (23.1%), mean age at onset (49.6 years), age at death (66.6 years) and duration of disease (16.7 years) were slightly higher than previously reported.

**Conclusion:**

HD did not experience true temporary variations in prevalence, incidence or mortality over 23 years of post-molecular testing in our population. Ascertainment bias may largely explain the worldwide heterogeneity in results of HD epidemiological estimates. Population-based rare diseases registries are valuable instruments for epidemiological studies on low prevalence genetic diseases, like HD, as long as they include validated data from multiple HIS and genetic/family information.

## Background

Huntington disease (HD) is a rare, autosomal dominant neurodegenerative disorder caused by the abnormal expansion of a CAG repeat sequence in the Huntingtin (*HTT*) gene. An expansion of 36 or more CAGs can lead to the disease, with earlier onsets associated with longer CAG repeats. HD is characterised by progressive motor, cognitive and/or psychiatric dysfunction, with onset typically occurring in the fourth decade of life [1].

The discovery of the mutation causing the disease in 1993 enabled unambiguous genetic testing, having a profound effect in the ascertainment of HD cases. Since then, multiple studies and methodologies have aimed to estimate its prevalence across different populations, displaying a highly variable HD distribution. Although HD is universal, it presents notable geographical differences, with the highest prevalence rates in western European origin populations and the lowest in Asian and African populations [2–5]. More recently, some studies among Caucasians have reported a substantial increase in prevalence, incidence and/or mortality rates, which might indicate a time variation in HD epidemiology [6–10]. However, whether it is, in fact, a true trend or secondary to an improved ascertainment process in post molecular years has not been fully investigated.

Another factor that may contribute to the variation in results among HD studies is the validity of different sources of ascertainment. Because of the rarity of the disease, most epidemiologic studies use administrative databases or health information systems (HIS) to identify HD cases. Nevertheless, classification and coding systems in current HIS are frequently nonspecific, which may result in a lack of completeness and accuracy of HD diagnosis. In parallel, population-based registry/surveillance registries are considered key instruments to estimate incidence and prevalence rates, temporal trends and geographical distribution of low prevalent diseases [11]. Given that data sources are potential windows for ascertainment bias, selection of datasets and diagnostic validation seem critical to maximize the quality of registries and their potential success as valuable resources for epidemiological research studies.

The Population-based Rare Diseases Registry of Navarre (RERNA) is an on-going registry, created in 2013 [12], with a specific registration protocol that includes: (a) extraction of “potential cases” from all available HIS; (b) comparison of cases through health identification codes and elimination of duplicates, (c) validation of diagnosis based on the criteria for each disease, (d) codification of validated diagnosis, (e) registration of socio-demographic variables of “confirmed cases”, and f) review of vital status and place of residence.

In addition to RERNA, Navarre counts with a clinical/genetic HD reference centre that provides services for HD patients and their families and collaborates in multicentre, multinational HD research studies. Our group has previously estimated the incidence and prevalence of HD in Navarre [13, 14]. In the present study, we aim to analyse the epidemiological trends of HD over a 27-year period in our community, and to examine the validity of different ascertainment sources, alone and in combination, used in population-based rare diseases registries (RDR).

## Methods

### Setting and study population

This study focuses on the population of Navarre, one of the 17 Autonomous Communities (AC) in northern Spain, with 647,554 inhabitants (50.51% women) in January of 2018, comprising 1.39% of the Spanish population [15].

The Spanish National Health System (S-NHS) is based on the principles of universality, free access, equity and fairness of financing, and is mainly funded by taxes [16]. Over 98% (637,683 individuals) of Navarre’s citizens have an individual health card with a unique 8-digit personal identification code (called CIPNA), which allows them to have access to the public health system. It contains information on birth date, sex and other socio-demographic conditions, and enables unique identification and matching of data among databases [17].

Systematic digital diagnostic coding in Navarre has not been evenly implemented for all HIS. Therefore, for the purpose of diagnostic code validation, we analysed data from the period 2000–2017 to ensure maximum ascertainment in all available data sources. Data for the epidemiology study included cases ascertained during a 27-year period, from January 1991 to December 2017. The study was approved by the Navarre Ethical Committee of Clinical Research.

### Sources of ascertainment

A. **Minimum Basic Data Set at Hospital Discharge (MBDS)**

The MBDS is a mandatory registry for all hospitals in Spain (both public and private) which links administrative data with clinical diagnoses. Medical diagnoses are encoded using the International Classification of Diseases (ICD): the Clinical Modification of its ninth revision (ICD-9-CM) until 2015, and the Spanish Clinical Modification of its version 10 (ICD-10-ES) thereafter [18, 19]. For this study, all episodes containing 333.4 (from 2000–2015) and G10 (from 2016–2017), as primary or supplementary diagnostic codes, in the Navarre’s MBDS were identified as potential HD cases.

B. **Electronic Clinical Records in Primary Care (ECRPC)**

The ECRPC is implanted in all Spanish regions and currently provides an on-going population-wide data source, as Primary Health Care is the first and most frequent point of contact between the population and the S-NHS [20]. In Navarre, primary care episodes are coded as per the International Classification for Primary Care (ICPC) issued by the WONCA [21]. These codes are not specific for rare diseases, but Navarre’s ECRPC includes additional literal descriptors for each ICPC code, some of which are specific for a rare disease or for a group of rare diseases. For the purpose of this study, the specific descriptor “Huntington disease, chorea” was used to identify potential HD cases during the study period.

C. **Temporary Work Disability Registry (TWDR)**

Workers who require a sick leave are given a temporary work disability initiation form, which entitles them to receive compensation payments from the Ministry of Work. Every temporary work disability episode has assigned an ICD-9-CM diagnostic code according to the cause reported by the primary care physician [22]. For this study, all temporary work disability episodes containing 333.4 code were selected from the Navarre’s TWDR during the period 2000–2017.

D. **Mortality Statistics (MS)**

Regional Health Ministries are in charge of the process of coding and registering the health variables of the deaths, including, the underlying cause of death (UCD) and, since 2014, the contributing cause of death (CCD) that have occurred in their territory [23]. The tenth revision of ICD coding system was adopted by the World Health Organization in 1989, and implemented in the Spanish MS as of 1999 [24]. For this study, mortality records from Navarre, containing G10 code (both UCD, and CCD since 2014) during 2000–2017 were identified.

E. ** Medical Genetics Centre (MGC)**

Navarre has a public reference MGC, located at the tertiary-level public hospital of the AC. Since 1991, patients with clinical signs compatible with HD and their relatives are referred to the MGC for assessment, counselling and molecular testing, when appropriate, following the HD guidelines for genetic testing [25, 26] and the pertinent signed informed consent. CAG repeat lengths are determined using PCR amplification assays with fluorescently labelled primers flanking the CAG repeat sequence [27]. The fragment size is determined by capillary electrophoresis with 3500 Genetic Analyzer (Applied Biosystems) and GeneMapper Software 5.

Demographic, clinical, family history and genetic data are collected and recorded in a disaggregated format, assigning an independent genetic family number that links to the CIPNA. Information on age at onset, age at diagnosis, parental origin of the disease, origin of family ancestors and, at least, three-generation family history is regularly obtained and revised at follow-up visits. The MGC is a site research centre for collaborative HD studies (Registry and Enroll-HD), with yearly follow-up evaluation of participants.

### Validation and diagnostic criteria

Case validation was performed using information from medical records and the clinical assessment of a neurologist and a clinical geneticist, both experts in HD. A detailed chart review was carried out and pertinent information was extracted from each chart. Pedigrees were also analysed to ascertain secondary cases, defined as symptomatic relatives who were not seen in the clinic, but were reported by family members as having signs compatible with HD.

Patients were diagnosed of HD if they fulfilled one of the following inclusion criteria: (1) Individuals with neurocognitive signs compatible with HD and a genetic test result of > 35 CAG repeats in the *HTT* gene; (2) Individuals showing neurocognitive signs compatible with HD, without a genetic test result available and with a genetically confirmed HD maternal or paternal family history.

Date at diagnosis was defined as that in which symptomatic patients were clinically diagnosed with HD or when a positive genetic test result (> 35 CAG repeats) was obtained. Patients who underwent presymptomatic testing and became symptomatic within the study period were also included, setting the diagnosis date as that of disease initiation.

### Epidemiology and demographic estimates

Point prevalence of HD was calculated annually using the number of HD symptomatic individuals per 100,000 inhabitants, resident in Navarre, on the 31st of December. Age- and sex-specific prevalence was estimated for the 31st of December, 2017. Incidence and mortality rates were defined as the number of newly diagnosed symptomatic HD cases and of HD-registered deaths, respectively, per 100,000 inhabitants per year. Mean annual incidence and mortality rates per 100,000 were analysed for three periods: 1991–1999, 2000–2008 and 2009–2017. For annual age-adjusted mortality rates, we used the 2013 European Standard Population as reference [28]. Overall trends were analysed for the three epidemiologic indicators.

### Statistics

Results were summarised using descriptive statistics, such as mean and standard deviation, frequencies and proportions. Positive predictive values (PPV) and 95% confidence intervals (95% CI) were estimated for each source of ascertainment as the fraction of HD cases that fulfilled the HD diagnostic criteria, or true positives (TP), with respect to all potential HD cases: TP and false positives (FP). Sensitivity and 95% CI were estimated as the fraction of confirmed HD cases identified by each source, with respect to the total number of HD individuals ascertained in the study (for MBDS, ECRPC, TWDR or MGC), or to the total deceased HD patients (for MS). Change-points, slopes and average annual per cent changes (AAPC) were assessed by Joinpoint regression, annually for prevalence and biannually for incidence and mortality.

## Results

### HD case ascertainment (HIS and MGC)

**Fig. 1:**
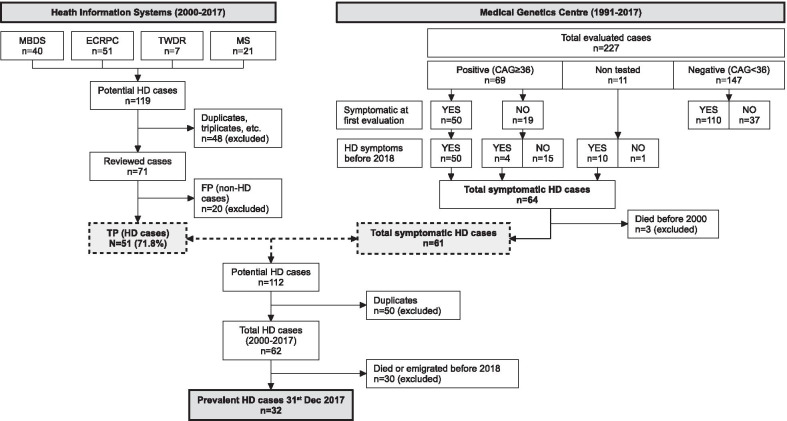
Flow diagram depicting the identification of HD cases on the 31st of December, 2017, from Navarre’s Health Information Systems (2000–2017), and from the Medical Genetics Centre (1991–2017), including the validation process. *MBDS* Minimum Basic Data Set at hospital discharge, *ECRPC* Electronic Clinical Records in Primary Care, *TWDR* Temporary Work Disability Registry, *MS* Mortality Statistics, *n* number of cases, *FP* false positives, *TP* true positives, *HD* Huntington’s Disease

HIS captured a total number of 119 potential HD cases between 2000 and 2017: 40 from MBDS, 51 from ECRPC, 7 from TWDR, and 21 from MS. Forty-eight of them (40.3%) were identified in more than one source, and duplicates were excluded from the analysis. The remaining 71 potential HD cases were reviewed to verify the diagnosis. Fifty-one (71.8%) were confirmed as TP HD cases and 20 (28.2%) were ruled out and classified as FP. Of these, eight were incorrectly coded (50% with unspecified chorea), 10 had negative genetic test results, and two had a positive family history but the presence of clinical signs could not be confirmed. A flow diagram of the identification and validation process of potential cases with HD is illustrated in Fig. 1.

From 1991 to 2017, a total of 227 individuals with clinical signs compatible with HD and/or family history of this disease, were evaluated at the MGC. Of them, 147 tested negative (110 symptomatic and 37 asymptomatic), 69 tested positive (50 symptomatic and 19 asymptomatic) and 11 did not get or want to be tested (10 symptomatic and one asymptomatic). Before 2018, four of the 19 asymptomatic positive cases showed neurocognitive signs, giving a total number of 64 HD manifest cases during the period (53.1% women). Three of them died before 2000. These findings are illustrated in Fig. 1.

### PPVs and sensitivity

For the period 2000–2017, 62 HD cases were ascertained combining HD cases notified by MGC and/or captured by the explored HIS. Of them, 29 were registered deceased and one emigrated.

**Fig. 2:**
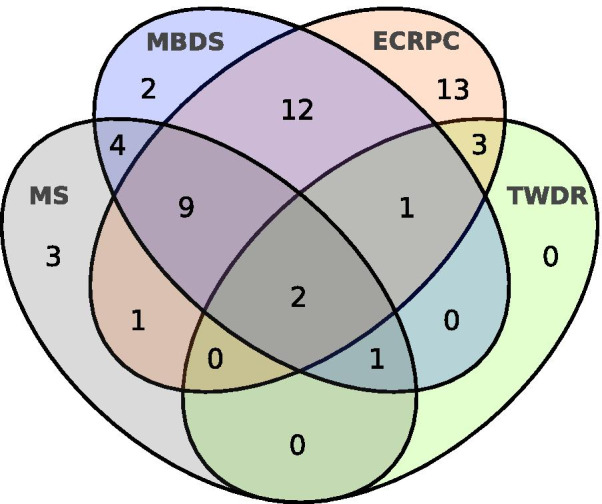
Venn diagram showing the overlapping HD cases of the Health Information Systems

The overall PPV for the combined HIS was 71.8% (95% CI: 59.7, 81.6). All FP cases were captured by only one source: nine by MBDS, 10 by ECRPC, none by TWDR and one by MS, yielding a PPV (95% CI) of 75.5% (61.2, 88.6), 80.4% (66.5, 89.7), 100% (56.1, 100) and 95.2% (74.1, 99.8), respectively (Table 1). ECRPC had the highest number of “unique to source” cases, in contrast to TWDR, with none. Eighteen cases (35%) were identified in a unique source and only two by all explored sources (4%). A Venn diagram illustrating HD confirmed cases and their overlap in the different HIS is presented in Fig. 2.

**Table 1:** Potential HD cases, true positives (TP), false positives (FP), positive predictive value (PPV) and sensitivity of all sources of ascertainment

Source of ascertainment	Potential HD cases	TP	FP	PPV (95% CI)	Sensitivity (95% CI)
MBDS	40	31	9	75.5% (61.2, 88.6)	50.0% (37.2, 62.8)
ECRPC	51	41	10	80.4% (66.5, 89.7)	66.1% (52.9, 77.4)
TWDR	7	7	0	100% (56.1, 100)	11.3% (5.0, 22.5)
MS	21	20	1	95.2% (74.1, 99.8)	69.0% (49.1, 84.0)
MGC	61	61	0	100% (92.6, 100)	98.4% (90.2, 100)

*MBDS* minimum basic data set at hospital discharge, *ECRPC* electronic clinical records in primary care, *TWDR* Temporary Work Disability Registry, *MS* mortality statistics, *MGC* Medical Genetics Centre

Sensitivity (95% CI), estimated for all combined HIS, was 82.2% (70.1, 90.4), with great variation among them: 50% (37.2, 62.8) for MBDS, 66.1% (52.9, 77.4) for ECRPC, 11.3% (5, 22.5) for TWDR, and 69% (49.1, 84) for MS (Table 1).

### Prevalence, incidence and mortality rates

A total of 80 HD cases were identified between 1991 and 2017, corresponding to 42 families. Of them, 69 cases were genetically confirmed, while 11 patients were diagnosed based on a genetically positive HD first-degree relative and the manifestation of neurological signs. On average, three new HD positive cases were identified per year throughout the study period (1991–2017), 2.3 symptomatic and 0.7 asymptomatic (Table 2), showing an increasing trend of pre-manifest testing in detriment of the symptomatic.

**Table 2:** Sex-, age- and period-specific estimates of prevalence and incidence of Huntington disease (HD) in Navarre, for the period 1991–2017

	Number of HD cases			Prevalence		Incidence	
	Total	Symptomatic	Asymptomatic (n)^a^	Total prevalent cases	Point prevalence rate per 100,000 (95% CI)	Total incident cases	Annual incidence rate per 100,000 (95% CI)
Total	80	61	19	65		63	0.40 (0.31, 0.50)
*Sex*							
Men	38 (47%)	26	12 (4)	15 (47%) ^b^	4.68 (2.31, 7.05)^b^	29 (46%)	0.37 (0.25, 0.52)
Women	42 (53%)	35	7	17 (53%) ^b^	5.20 (2.73, 7.67)^b^	34 (54%)	0.43 (0.30, 0.59)
*Age (years)*							
≤ 20	4	1	3 (1)	0^b^	0^b^	1	0.03 (0, 0.16)
21–40	22	8	14 (2)	4^b^	2.64 (0.05, 5.22)^b^	10	0.19 (0.10, 0.35)
41–60	28	26	2 (1)	10^b^	5.11 (1.94, 8.27)^b^	26	0.63 (0.42, 0.91)
> 60	26	26	0	18^b^	11.53 (6.20, 16.85)^b^	26	0.78 (0.52, 1.12)
*Period*							
1991–1999	26	23	3 (2)	31^c^	5.70 (3.69, 7.71)^c^	22	0.46 (0.30, 0.69)
2000–2008	28	21	7 (1)	32^d^	5.07 (3.31, 6.83)^d^	21	0.39 (0.25, 0.59)
2009–2017	24	15	9 (1)	32^b^	4.94 (3.23, 6.65)^b^	20	0.35 (0.22, 0.53)

^a^Number of HD presymptomatic cases that became symptomatic throughout the study period

Point prevalence dates: ^b^31/12/2017, ^c^31/12/1999, ^d^31/12/2008

**Fig. 3:**
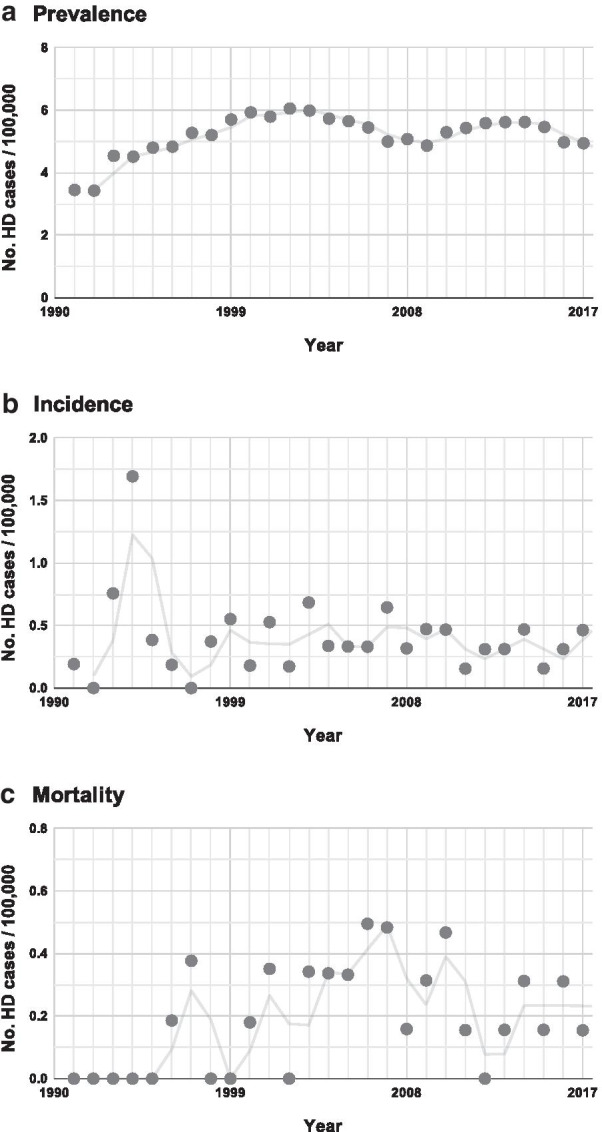
Population-adjusted HD epidemiologic trends for the period 1991–2017 in Navarre. **a** Point prevalence, calculated for the 31st of December of each year, **b** annual incidence, and **c** annual mortality

There were 65 HD manifest cases (54% women) during 1991–2017, which gave an estimated prevalence per 100,000 (95% CI) of 5.7 (3.69, 7.71) for 31/12/1999, 5.07 (3.31, 6.83) for 31/12/2008 and 4.94 (3.23, 6.65) for 31/12/2017 (Table 2). Of them, 63 were incident throughout the study period, 1991–2017. By 9-year intervals, 1991–1999, 2000–2008 and 2009–2017, mean annual incidence rates per 100,000 (95% CI) were 0.46 (0.30, 0.69), 0.39 (0.25, 0.59) and 0.35 (0.22, 0.53), respectively (Table 2). A total of 32 deaths were recorded throughout the study, with mean annual rates per 100,000 (95% CI) for each 9-year period of 0.06 (0.02, 0.17), 0.3 (0.18, 0.49) and 0.23 (0.13, 0.38), respectively. Figure 3 shows the annual rates of prevalence, incidence and mortality during 1991–2017.

**Fig. 4:**
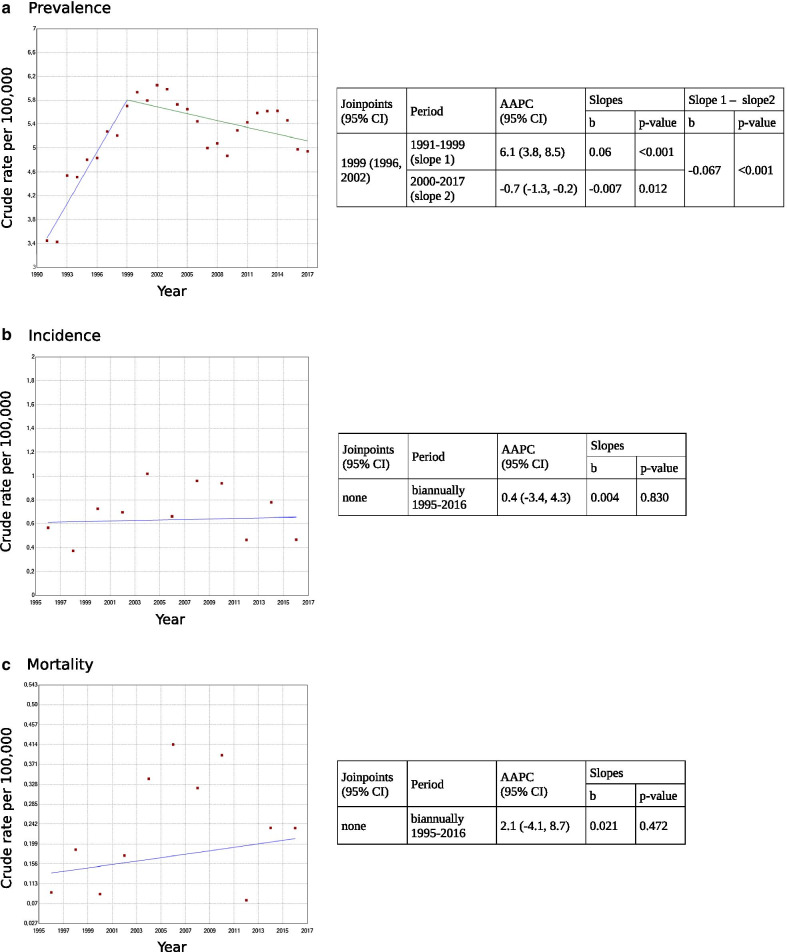
Huntington disease prevalence **a**, incidence **b** and mortality **c** trends, change points, average annual per cent changes (AAPC) and slopes using a Joinpoint regression model

Regarding evolution, Joinpoint analysis for prevalence showed a change-point in 1999 (95% CI: 1996–2002), with an increasing slope until 1999 at an AAPC of 6.1 (95% CI: 3.8, 8.5) and a slightly decreasing slope from 1999 onwards with AAPC of – 0.7 (95% CI: − 1.4, − 0.2) (Fig. 4). Incidence and mortality experienced unstable annual rates during the first period (1991–1999), showing a distinctive incidence peak in 1993–1994 and a very low number of deaths, respectively. However, from the biennium 1995–1996 onwards, both annual rates remained stable, with no relevant changes. Joinpoint results for incidence and mortality during this 23-year period did not show trend changes or significant slopes, with AAPC (95% CI) of 0.4 (− 3.4, 4.3) and 2.1 (− 4.1, 8.7), respectively. Overall annual incidence and mortality rates per 100,000 (95% CI) during 1995–2017 were 0.36 (0.27, 0.47) and 0.23 (0.16, 0.32), respectively. Age-adjusted mortality rate was 0.24 per 100,000 (95% CI: 0.09, 0.76).

**Fig. 5:**
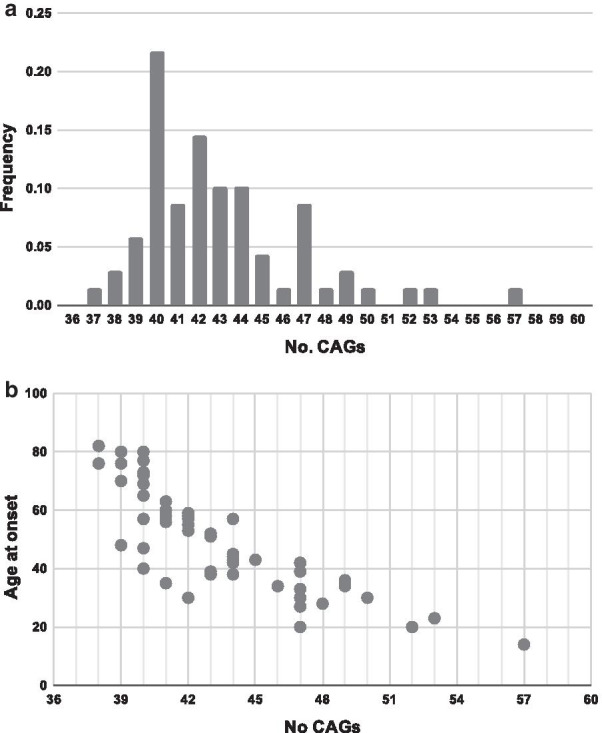
**a** Histogram of the number of CAGs of the *HTT* gene (n = 69). **b** Plot representing the disease onset by number of CAG of Huntington patients in Navarre's cohort (n = 54)

The mean number of CAG repeats in the *HTT* gene was 43 ± 3.8 (range 37–57 CAGs, n = 69). HD cases had a mean age at onset of 49.8 ± 18.8 years (range 14–82, n = 63) (Fig. 5) and a mean age at incidence of 55.7 ± 16.5 years (range 15–85, n = 63). Juvenile forms of HD represented 6.2% of cases, and 23.1% showed symptoms after 60 years of age (late-onset HD). Mean CAG length for late-onset cases was 39.5 ± 0.91 (range 38–41, n = 12). Across the studied period, 32 patients were deceased at an average age of 66.6 ± 17.1 years (range 27–90) and, overall, the disease duration was 16.7 ± 8.1 years (range 4–39, n = 31). Suicide was the cause of death in 6.3% of cases.

## Discussion

The present study is the first to validate the accuracy and sensitivity of the main HD diagnostic codes in different routinely collected health-care datasets, alone and in combination, using medical records as the gold standard. We also provide unbiased HD epidemiological estimates and trends from 1991 to 2017, in a well-defined geographic region in northern Spain, Navarre, using supplementary clinical, genetic and family data from the genetic reference centre of the region.

Approximately 2/3 of HD cases identified across all four HIS were confirmed by review of clinical/genetic records, with individual dataset PPVs ranging from 76% for hospital discharges data to 100% for temporary working leave information. PPVs for primary care and mortality data codes were 80% and 95%, respectively. These figures are within the range of those reported for other neurodegenerative diseases, such as Parkinson Disease [29], Charcot Marie Tooth [30], dementia [31], Guillain-Barré syndrome [32] or Duchenne/Becker muscular dystrophy [33].

Sensitivity, however, was more diverse among datasets (from 11% in TWDR to 69% in MS). Primary care data was a more valuable resource for HD case ascertainment than hospital discharge electronic records. Combination of both sources identified 77% of all HD cases with a PPV of 72%. TWDR and MS databases presented the highest PPV of explored HIS (100% and 95%, respectively); on the contrary, they had lower capacity to identify HD cases. Similar variation in sensitivity of routinely collected health-care data has also been observed for Parkinson disease [29]. We are not aware, however, of analogous studies for HD or other rare diseases with genetic diagnosis; therefore, further comparative analysis of our results is not yet feasible.

Epidemiologic findings show that, on 31st December 2017, the prevalence of manifest HD in Navarre was 4.94 per 100,000, with an average annual incidence rate of 0.36 per 100,000 inhabitants (1995–2017). These estimates are within the contemporary European range [3, 34] and in concordance with our general population CAG repeat length distribution (data not shown). Prevalence, however, is lower than that reported in the United Kingdom [7] or Ireland [35], higher than in some northern European countries like Finland [36] or Iceland [37], and in line with other southern European populations [38–41]. Other post molecular studies carried out in Spain have also reported comparable HD prevalence figures (4.6 and 4.0, per 100,000 in Asturias and Murcia, respectively [42, 43]; in contrast, lower prevalence was observed for Balearic Islands (2 per 100,000) [43]. It is likely, however, that this low prevalence rate reflects incomplete ascertainment, given the limitations in data sources and length of the study period (four years). Similarly, we observed a higher mean annual adjusted mortality rate (0.24 per 100,000 during 1991–2017) than that previously reported in Spain for an overlapping period (0.08 and 0.15 per 100,000 in 1991 and 2013, respectively) [9]. Worldwide, however, HD mortality remains understudied, with a few, mainly pre-molecular reports, showing comparable rates to ours in the United States (0.23/100,000) [44] and lower in Austria (0.13/100,000) [45].

It is generally accepted that availability of direct HD testing increased ascertainment of cases, by the identification of patients with unknown HD family history, which occurs in approximately 10–16% of cases [7, 13]. Consequently, HD overall prevalence and incidence estimates are higher than in pre-testing decades [7, 8, 34, 36]. Nevertheless, there is still wide geographic variation among studies, which cannot be fully explained by the population genetic background, including the pool of intermediate CAG repeat alleles and *HTT* haplotypes. Moreover, there is some evidence of a potential trend of increasing HD rates in some populations [7, 10]. However, interpretation of results is controversial, as most studies differ in demographic characteristics, case-sources and case-ascertainment methods. Our study demonstrates that prevalence, incidence and mortality rates of HD in our population did not experience a true increase over time, showing stable estimated trends over the last 23-years of post-molecular HD testing. Interestingly, prevalence rates showed an increasing trend during 1991–1999, while incidence was slightly higher than in the following years (2000–2017). As shown in Fig. 3, annual incidence experienced a distinctive peak in 1993–1994, suggesting that the excess of incident cases in the first study period is, most likely, a consequence of the availability of direct HD testing which allowed the identification of previously suspected, but undiagnosed, HD cases. It resulted in a 6.1% average increase in annual prevalence until 1999, followed by a very slight decrease (0.7%) thereafter. The number of prevalent cases did not vary significantly over this period, but the total population experienced a 20% increase since 1991 (data not shown). It is, therefore, conceivable that demographic changes in the population might have contributed to slightly decrease the prevalence trends. With respect to mortality, no deaths from HD were recorded before 1996, but improvement in HD ascertainment resulted in stable annual rates therafter. Very low mortality rates have been also observed in Spain in the late 1980s with increasing trends until 2013 [9].

Most epidemiologic HD studies use health-care databases as the main source of ascertainment. Our study strongly suggests that ascertainment bias may be an important factor that could explain, at least in part, geographic and temporary differences in reported HD prevalence and incidence rates. According to our results, individual hospital discharges and primary health-care datasets might miss 30–50% of prevalent HD cases and include over 20% of non-HD patients. Misclassification of cases mainly involved: a) under-ascertainment of late-onset patients showing neurocognitive signs commonly seen in other relatively frequently diseases, like Alzheimer disease, obsessive–compulsive disorder and other dementias and psychiatric illnesses, and, b) inclusion of asymptomatic mutation carriers and negative HD family members. In the present study, 47% of FP in hospital and primary care datasets were asymptomatic members of HD families, either with unknown or negative genetic testing. In this regard, it is worth mentioning that, although uptake of HD predictive testing is overall low [46], the expectation for better medical interventions, including the availability of preconceptiol diagnosis and potentially promising new treatments, may result in a temporary increase of genetic testing in asymptomatic individuals. We, in fact, observed that over one-third of all HD positive cases identified during the most recent study period (2000–2017), were asymptomatic, a proportion three times higher than that during 1991–1999. It would be interesting to investigate this issue in other and larger populations and its possible effect as a potential bias in HD ascertainment.

To overcome the above-mentioned limitations of individual HIS ascertainment, several studies used multiple sources of information, yielding higher true prevalence HD rates [8, 10]. As a counterpart, however, these studies are more likely to double/triple-count a relatively large number of individuals. In our analysis, 43% of potential HD cases were included in both primary care and hospital databases, and 64.5% would be double-counted when combined with mortality dataset. Consequently, minimizing overestimation of true HD prevalence/incidence in multiple sources ascertainment studies requires further highly time-consuming investigations, which may not be feasible when dealing with large populations. Finally, we also proved that the inclusion of genetic and family data is a relevant source that adds high validity to case ascertainment. The MGC ascertained 14 ‘unique to source’ cases, corresponding to 23% of HD cases, and identified 65% of FP. We, therefore, conclude that population-based RDR are potentially a highly valuable instrument to conduct epidemiological studies on low prevalence, like HD, providing the inclusion of multiple health and administrative validated sources of information, in conjunction with genetic and family data.

As expected, demographic characteristics of HD patients in Navarre were similar to those reported in most Caucasian populations. We observed, however, some interesting differences. HD natural history seems to present with a wider range in the timing of initiation of signs, higher proportion of late-onset HD cases (23.1%), and longer overall survival than previously estimated [8, 38, 39, 41, 47–49]. This is most likely due to improvements in clinical and molecular HD ascertainment, which, in our population, resulted in the identification of a high proportion of cases with low-penetrant alleles (10%). Additional circumstances, such as better health-care interventions may have also contributed to increase quality of life and extended life expectancy.

The main limitation of the present study is the small population coverage and sample size of HD cases, given the low prevalence of the disease. In addition, variability in access to health-care systems and diagnostic coding specificity in different populations could limit the possibility of extrapolating our diagnostic code validation results to a national or international scale. We must mention, to this respect, that the annual adjusted mortality rate in our regional study was 40% higher (0.24 per 100,000) than the overall rate previously reported in Spain (0.15 per 100,000) using MS for an overlapping period (1991–2013) [9]. This difference is in concordance with our results on sensitivity of the national mortality dataset, supporting the value of this validation study. Finally, the strength of the present work lies in the study design, an HD population-based analysis of nearly three decades, with complete case ascertainment, using clinical and genetic data as reference standards.

## Conclusions

We present the first HD diagnostic code validation analysis for different HIS, and demonstrate that epidemiological estimates for this rare disease in Navarre do not show true temporary variations during the last decades of post molecular testing. Improved HD ascertainment may decrease heterogeneity among worldwide HD epidemiological studies and result in a higher identification of low-penetrant allele carriers that will widen the knowledge of the natural history of the disease.

## Data Availability

The datasets used and analysed during the current study are available from the corresponding author upon reasonable request.

## Abbreviations

95% CI: 95% Confidence intervals
AAPC: Average annual per cent changes
AC: Autonomous Communities
CCD: Contributing cause of death
ECRPC: Electronic Clinical Records in Primary Care
FP: False positives
HD: Huntington Disease
HIS: Health information systems
ICD: International Classification of Diseases
ICD-9-CM: International Classification of Diseases, Ninth Revision, Clinical Modification
ICD-10-ES: International Classification of Diseases, Tenth Revision, Clinical Modification (Spanish adaptation)
ICPC: International Classification for Primary Care
MBDS: Minimum Basic Data Set at Hospital Discharge
MGC: Medical Genetics Centre
MS: Mortality Statistics
PPV: Positive predictive values
RDR: Rare diseases registries
RERNA: Population-based Rare Diseases Registry of Navarre
S-NHS: Spanish National Health System
TP: True positives
TWDR: Temporary Work Disability Registry
UCD: Underlying cause of death
